# Gene Expression and Chromatin Modifications Associated with Maize Centromeres

**DOI:** 10.1534/g3.115.022764

**Published:** 2015-11-12

**Authors:** Hainan Zhao, Xiaobiao Zhu, Kai Wang, Jonathan I. Gent, Wenli Zhang, R. Kelly Dawe, Jiming Jiang

**Affiliations:** * Department of Horticulture, University of Wisconsin, Madison, Wisconsin 53706; † Department of Plant Biology, University of Georgia, Athens, Georgia 30602

**Keywords:** CENH3, centromere, gene expression, histone modification, nucleosome

## Abstract

Centromeres are defined by the presence of CENH3, a variant of histone H3. Centromeres in most plant species contain exclusively highly repetitive DNA sequences, which has hindered research on structure and function of centromeric chromatin. Several maize centromeres have been nearly completely sequenced, providing a sequence-based platform for genomic and epigenomic research of plant centromeres. Here we report a high resolution map of CENH3 nucleosomes in the maize genome. Although CENH3 nucleosomes are spaced ∼190 bp on average, CENH3 nucleosomes that occupied *CentC*, a 156-bp centromeric satellite repeat, showed clear positioning aligning with *CentC* monomers. Maize centromeres contain alternating CENH3-enriched and CENH3-depleted subdomains, which account for 87% and 13% of the centromeres, respectively. A number of annotated genes were identified in the centromeres, including 11 active genes that were located exclusively in CENH3-depleted subdomains. The euchromatic histone modification marks, including H3K4me3, H3K36me3 and H3K9ac, detected in maize centromeres were associated mainly with the active genes. Interestingly, maize centromeres also have lower levels of the heterochromatin histone modification mark H3K27me2 relative to pericentromeric regions. We conclude that neither H3K27me2 nor the three euchromatic histone modifications are likely to serve as functionally important epigenetic marks of centromere identity in maize.

Centromeric chromatin contains the histone H3 variant cenH3 (CENP-A in humans, CENH3 in maize)—a defining feature of centromeres (Henikoff *et al.* 2001). While this H3 variant makes up only a small percentage of the total H3 population in centromeres (Fukagawa and Earnshaw 2014), it plays a central role in recruiting other centromere proteins. The patterns and functional significance of other histone variants and histone modifications in centromeres are much less well understood, and are likely variable between organisms. For example, centromeres of the filamentous fungi *Neurospora crassa* have heterochromatic histone modifications (Smith *et al.* 2012), while centromeres of the fission yeast *Schizosaccharomyces pombe* have euchromatic histone modifications (Cam *et al.* 2005). Similarly, both euchromatic and heterochromatic histone modifications have been found in the centromeres of different plant and animal species (Sullivan and Karpen 2004; Guenatri *et al.* 2004; Yan *et al.* 2005; Ribeiro *et al.* 2010; Wu *et al.* 2011).

Centromeres contain both H3 and cenH3 nucleosomes (Blower *et al.* 2002). Immunofluorescence staining of animal centromeres has revealed the presence of distinct domains within centromeres, some with reduced cenH3 occupancy (reviewed by Fukagawa and Earnshaw 2014). Genome-wide mapping of sequences associated with cenH3 nucleosomes in several plant species revealed distinct cenH3-enriched and cenH3-depleted subdomains in individual centromeres (Yan *et al.* 2008; Gong *et al.* 2012; Gent *et al.* 2012). In rice centromeres, several euchromatic histone modification marks were associated with subdomains that are depleted of cenH3 nucleosomes. These subdomains also contain a low density of transcribed genes (Yan *et al.* 2005, 2006). The CENH3-binding domains of maize centromeres expand from ∼1.8 Mb to ∼3.6 Mb when the maize chromosomes are transferred into the genetic background of oat (Wang *et al.* 2014). Interestingly, the centromeres appeared to expand preferentially into flanking regions that lack genes. These results suggest that cenH3 chromatin is not compatible with transcription. Another striking character associated with centromeric chromatin is that cenH3 nucleosomes appear to protect less DNA from MNase digestion than canonical nucleosomes. The “point” centromeres of budding yeast *Saccharomyces cerevisiae* contain a single cenH3 nucleosome consisting of ∼120 bp centromeric DNA sequence (Krassovsky *et al.* 2012; Henikoff and Henikoff 2012). Similarly, the cenH3 nucleosomes in humans and rice protect ∼90–130 bp sequences from MNase digestion (Hasson *et al.* 2013; Zhang *et al.* 2013). In addition, cenH3 nucleosomes were found to be highly phased on the centromeric satellite repeats in both humans (Hasson *et al.* 2013; Henikoff *et al.* 2015) and rice (Zhang *et al.* 2013).

The maize genome has been sequenced based on a minimum tilling path of bacterial artificial chromosomes (Schnable *et al.* 2009). Several maize centromeres contain only limited amounts of the centromeric satellite repeats, and represent some of the best sequenced centromeres in multicellular eukaryotes (Wolfgruber *et al.* 2009). These well sequenced centromeres provide us a platform to profile chromatin modifications and RNA expression patterns associated with maize centromeres. We developed a high resolution genome-wide map of maize CENH3 nucleosomes based on Illumina sequencing of DNA samples prepared from chromatin immunoprecipitation (ChIP) using a maize anti-CENH3 antibody. This map has delineated fine boundaries of alternating CENH3-enriched and CENH3-depleted subdomains in seven of the 10 maize centromeres. We identified a set of 34 annotated genes in the centromeres. However, only 11 genes located in CENH3-depleted subdomains were transcriptionally active. These active genes were associated with euchromatic histone modification marks. In addition, maize centromeres lacked both euchromatic histone modifications (except for the regions associated with active genes) and H3K27me2, a mark of heterochromatin in maize. We demonstrate that deep CENH3 ChIP-seq datasets allow mapping of CENH3 nucleosome positioning and dynamics in maize.

## Materials and Methods

### MNase digestion, ChIP, and ChIP-seq

Ten-day-old maize (B73) seedlings grown in the greenhouse were collected and ground into fine powder in liquid nitrogen for nuclei extraction. The input chromatin for anti-CENH3 ChIP experiments was prepared from purified nuclei digested with 0.2 unit (U) and 5 U of micrococcal nuclease (MNase; Sigma-Aldrich, N5386_200U), respectively. ChIP experiments, including nuclei purification, followed published protocols (Zhang *et al.* 2012). Both ChIPed DNA and input DNA corresponding to 0.2 U and 5 U MNase trimmed chromatin input were used for ChIP-seq library preparation following published methods (Henikoff *et al.* 2011) to recover and sequence small DNA fragments. The DNA libraries were sequenced using the Illumina HiSequation 2000 platform. For MNase digestion of naked DNA, DNA was extracted from a B73 developing ear using a DNeasy Plant Mini Kit (Qiagen), and digested with MNase (New England Biolabs, #M0247S) as follows: a master mix consisting of 140 ng/μl DNA, 82 gel units/μl MNase, 150 ng/μl BSA in 1X micrococcal nuclease buffer was prepared then divided into five 150-μl aliquots on ice. All aliquots were simultaneously transferred to a 37° water bath then removed one at a time after 1, 4, and 8 min (at which time the bulk of DNA had been digested to fragments of less than 200 bp in length). The digested was purified and prepared for Illumina sequencing as described (Gent *et al.* 2014).

### Gene expression analysis using quantitative real-time PCR

RNA was extracted from 10-day-old maize (B73) seedlings using an RNeasy Plant Mini Kit (Qiagen, 74904) following the manufacturer’s instructions. RNA samples were treated with TURBO DNase (Ambion, AM1907), and reverse transcribed with SuperScript III (Invitrogen, 18080-051). The expression levels of a set of seven centromeric genes (Supporting Information, Table S1) relative to the *Actin* reference gene were validated by both semiquantitative real-time PCR (semiqRT-PCR) and quantitative real-time PCR (qRT-PCR) carried out on a Thermal Cycler (Bio-Rad, C1000) using rTaq DNA polymerase (Takara, R001A), and on MJ Research Opticon 2 (Bio-Rad Laboratories) using the SYBR Advantage qPCR Premix (Clontech, 639676), respectively. Annealing temperature was set to 60°. SemiqRT-PCR and qRT-PCR primers are provided in Table S1.

### Sequence mapping and identification of CENH3-binding domains

Raw sequencing reads were trimmed by Cutadapt (Martin 2011) to remove low quality nucleotides (with quality score less than 30) and adapters. Pairs with reads of less than 26 bp in length for either read after quality and adapter trimming were discarded. Reads were mapped to the B73 reference genome (RefGen_v3, http://plants.ensembl.org/Zea_mays) by BWA-MEM using default parameters (Li 2013). Only uniquely-mapped reads were retained for subsequent analyses (mapping quality of at least 20 and perfect matches) (Li *et al.* 2009). CENH3 ChIP-seq data from the 0.2 U and 5 U MNase-digested samples were analyzed independently. To analyze CENH3 enrichment, each chromosome was divided into 10-kb nonoverlap windows. The number of ChIP-seq reads in each window was normalized using input reads. CENH3 enrichment of each window was determined by comparing the number of reads from ChIPed and input data. CENH3-binding domains were identified by comparing the ChIPed and input data using SICER (Zang *et al.* 2009), which is designed to identify ChIP-enriched signals that tend to form specific subdomains. The parameters for SICER included a window size of 1000 bp, gap size of 3000 bp, effective genome fraction 0.7, redundancy threshold 1, fragment size 150 bp, and false discovery rate (FDR) 0.05. To determine the sizes and positions of centromeres, we merged CENH3-binding domains that were separated by less than 100 kb to eliminate the gaps that arise because of insufficient mapping at highly repetitive regions. Small CENH3-binding subdomains of less than 30 kb were discarded.

### Transcriptome, histone modification, and CENH3-depleted subdomains

Transcriptome (SRR445245 and SRR445382) and ChIP data of H3K27me3, H3K36me3, H3K4me3 and H3K9ac (GSE1528) (Wang *et al.* 2009), and H3K27me2 (Gent *et al.* 2014) (SRR1584368 and SRR1584369) were downloaded from NCBI (http://www.ncbi.nlm.nih.gov/). Transcriptome reads were mapped using TopHat (Trapnell *et al.* 2009), and ChIP reads were mapped using BWA-MEM with default parameters (Li 2013). Only uniquely mapped reads were retained. Gene expression in centromeres was analyzed by Cufflinks (Trapnell *et al.* 2010) using gene annotation AGPv3.21 (http://plants.ensembl.org/Zea_mays) with default parameters. We used MACS2 (-B –broad –g 740000000–trackline) (Zhang *et al.* 2008) and SICER (window size 200 bp, gap size 400 bp, effective genome fraction 0.4, redundancy threshold 1, fragment size 150 bp, and FDR 0.05) to identify histone modification peaks in centromeres, and retained peaks that were consistently discovered by both algorithms for further analysis. To analyze the distributions of each histone modification, the genome was divided into 100-kb windows. Mappable regions within the windows were determined by mapping simulated reads to genome sequence. Read density was calculated as the number of reads in the mappable regions of each window.

To identify CENH3-depleted subdomains in the centromeres, we divided the B73 genome into 10-kb windows and counted uniquely-mapped reads from ChIP and input (0.2 U and 5 U libraries combined). ChIP read counts were then normalized by input to calculate enrichment for each 10-kb window. Read count distribution of the input data were generated. For each window, read count of the ChIPed data was tested against the input distribution. Windows with a *p*-value less than 1 × 10^−5^ were considered as CENH3-depleted subdomains. Some regions may have fewer reads due to inefficient mapping at repetitive regions rather than paucity of CENH3. To overcome a potential mapping bias caused by repetitive DNA sequences, we generated 150-bp reads randomly from the B73 genome, and aligned these reads to the genome to identify mappable 10-kb windows in centromeres. CENH3-depleted subdomains overlapped to mappable regions were retained. Portions of CENH3-depleted subdomains that overlapped with unmappable regions were discarded. In addition, we discarded CENH3-depleted subdomains located at the ends of centromeres (less than 200 kb to the ends of centromere boundaries) as these regions may not represent CENH3 depletion.

To analyze the relationship between histone modification and maize centromere expansion in oat, we divided the expanded regions of *Cen2*, *Cen5*, *Cen9*, and *Cen10* into 1-kb windows and calculated read density of H3K4me2, H3K36me3, H3K9ac, H3K27me3, and H3K27me2 in each window. Read densities were then normalized by the mappability of each window. Read densities of nonexpanded regions were calculated in the same manner. The significant differences of each histone modification between expanded regions and nonexpanded regions were determined by bootstrap resampling of read densities from expanded regions and nonexpanded regions.

### Nucleosome positioning and phasing on *CentC*

We analyzed the nucleosome positioning using nucleR (Flores and Orozco 2011) with slight modification. Reads mapped to centromeres were extracted and trimmed to 40 bp around the center of each pair. Read depth of each position in centromeres was then determined by BEDtools (Quinlan and Hall 2010). Read depth was analyzed by nucleR with parameter “pcKeepComp” set to 0.01. “pcKeepComp” was set to 0.02 to identify closely located peaks on *CentC* arrays. We annotated the maize satellite repeat *CentC* using CENSOR (-nofilter –s –bprm ‘-filter = none’) (Kohany *et al.* 2006). The consensus *CentC* sequence was generated by CAP3 (-j 200 -s 1000 -o 100 -p 80) (Huang and Madan 1999) using ∼9850 (82% of total *CentC* monomers) *CentC* monomers. Paired-end reads were merged by FLASH (-M 100 –z –t 1) (Magoc and Salzberg 2011), and then mapped to the B73 genome and a *CentC* tetramer using BWA-MEM (Zhang *et al.* 2013). Phasing of CENH3 nucleosomes was analyzed using previously described methods (Zhang *et al.* 2013).

### Data availability

The CENH3 ChIP-seq sequencing data are available from NCBI sequence read archive (SRA) under accession SRP057754, the MNase-digested maize genomic DNA sequencing data are under accession SRP049952 (http://www.ncbi.nlm.nih.gov/sra).

## Results

### High-resolution mapping of CENH3-binding domains of maize centromeres

**Figure 1:**
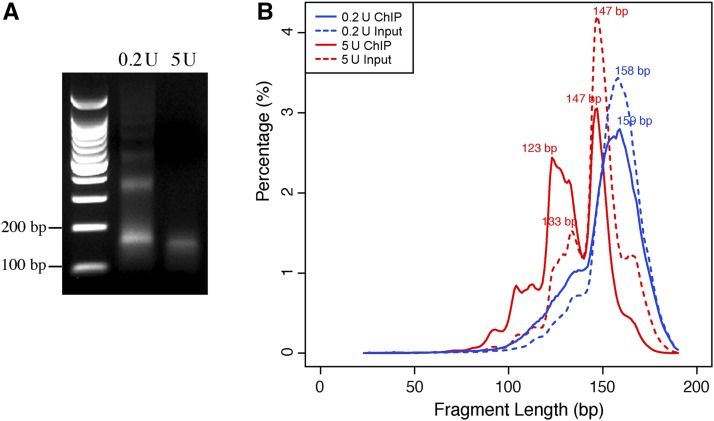
Chromatin immunoprecipitation (ChIP) and sequencing of maize centromeres. (A) Agarose gel electrophoresis of DNAs isolated from maize chromatin digested by 0.2 U and 5 U micrococcal nuclease (MNase), respectively. Libraries were developed from both ChIPed and input DNA samples of both chromatin samples. (B) Distribution of fragment lengths from 0.2 U and 5 U ChIPed and input sequencing libraries. The *x*-axis represents the length of fragments (bp). The *y*-axis represents the percentage of reads in specific length.

We conducted two independent ChIP experiments using anti-CENH3 antibody (Zhong *et al.* 2002). Chromatin isolated from B73 seedling tissue was digested by either 0.2 U or 5 U of MNase. The sample treated with 5U MNase was nearly fully digested (Figure 1A). Mono-nucleosomal DNA fragments of approximately 100–200 bp were gel-purified from both samples for library construction. A total of four libraries, two from ChIPed DNA and two from input DNA, were sequenced using the Illumina HiSequation 2000 platform, producing a total of 283 million 100-bp paired-end reads from the four libraries (Table S2). Sequence reads were mapped to maize B73 genome (version 3) (Schnable *et al.* 2009). Only uniquely mapped reads with perfect matches to the B73 genome were retained for further analysis.

Approximately 23% of the uniquely-mapped reads from the 0.2 U and 11% from the 5 U libraries were mapped to the centromeres (Table S2), which represented 45-fold and 27-fold CENH3 ChIP sequence enrichments in centromeres compared to input. The ChIP-seq read density across the maize genome was highly correlated between the two libraries (*R* = 0.90, *p* < 2.2 × 10^−16^, Spearman’s rank correlation). The lengths of the uniquely-mapped sequence reads from 0.2 U ChIP and input libraries peaked at 158 bp and 159 bp, respectively, *i.e.*, longer than the 147 bp that would wrap the canonical nucleosome core particle. These results indicate that the 0.2 U chromatin sample was underdigested and most reads contained linker sequences. The lengths of the sequence reads from the 5 U ChIP and input libraries peaked at 147 bp, with two minor peaks at 123 bp and 133 bp, respectively (Figure 1B). The minor peaks were also clearly visible in the input, of which only a small percentage of the reads is expected to be derived from CENH3-containing nucleosomes. Thus, these smaller fragments are abundant independently of CENH3, but are particularly enriched by CENH3 ChIP.

We divided each chromosome into 10-kb nonoverlapping windows and calculated ChIP-seq read enrichment within each window. Unambiguous enrichment peaks were observed in centromeric regions of chromosomes 2, 3, 4, 5, 8, 9, and 10 (Figure S1), consistent with previous centromere mapping results (Wolfgruber *et al.* 2009; Wang *et al.* 2014; Gent *et al.* 2015). Several minor peaks were also observed in chromosomal arms (Figure S1), which may result from misassembly or misplacement of centromere-associated repetitive DNA sequences. We observed very narrow peaks in centromeres 6 and 7, and no unambiguous peak in centromere 1, suggesting that these three centromeres contain mainly highly repetitive DNA sequences. Indeed, fiber-FISH mapping revealed that both *Cen1* and *Cen7* contain megabase-sized arrays of the *CentC* satellite repeats (Jin *et al.* 2004). We excluded *Cen1*, *Cen6*, and *Cen7* in further analysis.

We used SICER (Zang *et al.* 2009) to identify CENH3-binding domains using the 0.2 U and 5 U ChIP-seq data, independently. The CENH3-binding regions identified by the two datasets were highly correlated (Table S3). The consensus CENH3-binding regions identified in both datasets were then considered as the functional centromeres. We considered the SICER-defined peaks of CENH3 enrichment as the centromere of each chromosome (Table 1). The CENH3-binding domains ranged from 0.93 Mb of *Cen4* to 1.88 Mb for both *Cen5* and *Cen8*. This range, however, may not accurately reflect the true variation of centromere size because highly repetitive sequences in the centromeres are either missing in the reference genome or/and the relevant ChIP-seq reads were not mappable. *Cen2* and *Cen5*, the two best assembled maize centromeres, spanned 1.82 and 1.88 Mb, respectively.

**Table 1:** The positions of the centromeres in maize (B73) genome

Centromere	Start (Mb)	End (Mb)	Length (Mb)
*Cen2*	93.49	95.31	1.82
*Cen3*	99.80	100.79	1.00
*Cen4*	105.36	106.29	0.93
*Cen5*	102.18	104.06	1.88
*Cen8*	49.07	50.95	1.88
*Cen9*	52.43	53.68	1.26
*Cen10*	50.16	51.80	1.65

*Cen1*, *Cen6*, and *Cen7* were excluded due to low mappability and/or poor assembly of sequences associated with these three centromeres.

### CENH3-depleted subdomains in maize centromeres

**Figure 2:**
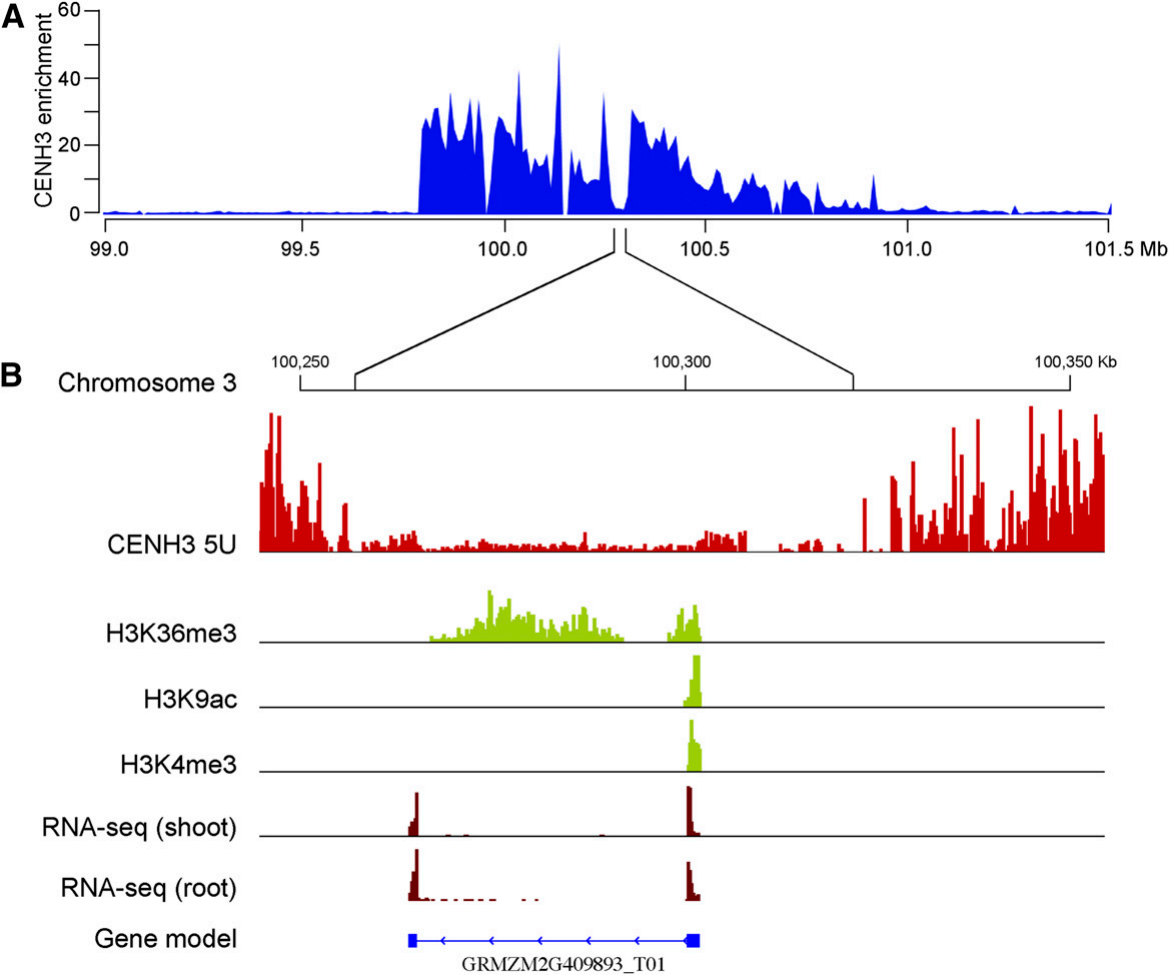
Position and structure of maize *Cen3*. (A) The position of *Cen3* was mapped between 99.80 Mb and 100.79 Mb. The *y*-axis represents fold enrichment of CENH3 ChIP-seq reads. (B) A CENH3-depleted subdomain (100.26–100.32 Mb) within *Cen3* is exemplified. This subdomain is mainly occupied by an active gene *GRMZM2G409893*. ChIP-seq reads from euchromatic histone modification marks H3K36me3, H3K9ac, and H3K4me3 within this subdomain were exclusively associated with the gene.

The CENH3 ChIP-seq reads were not evenly enriched in maize centromeres, which could be related to the lack of unique sequence in some regions in centromeres, and/or could indicate the existence of intermingled CENH3-enriched and CENH3-depleted subdomains as reported in several animals and plant species (Blower *et al.* 2002; Yan *et al.* 2008; Gong *et al.* 2012). To profile CENH3 binding in maize centromeres, we first determined each 10-kb window of each centromere as mappable or unmappable based on mapping 150-bp sequence reads that were generated computationally from the maize genome. Unmappable 10-kb windows in the centromeres were excluded because the CENH3-binding in these regions cannot be determined. A total of 1350 Mb mappable regions were identified in maize genome, including 6.5 Mb in centromeres, which comprised 53% of the identified centromeric sequences. We then divided the centromeres into 10-kb windows, and determined the CENH3 read enrichment of each window by comparing CENH3 ChIP-seq data to the input data (see *Materials and Methods*). We identified a total of 846 kb of CENH3-depleted subdomains (see *Materials and Methods* for definition of a CENH3-depleted subdomain) within the seven maize centromeres, which consisted of 13% of mappable centromeric regions (Table 2). The CENH3-depleted subdomains were interspersed among CENH3-enriched regions in centromeres (Figure 2). The percentages of CENH3-depleted subdomains were different among centromeres, ranging from 9.9% (*Cen5*) to 26.2% (*Cen3*) of the mappable sequences (Table 2). For this and all subsequent analyses, we combined both the 0.2 U and 5 U ChIP datasets into a single set.

**Table 2:** Summary of CENH3-depleted subdomains and histone modifications in maize centromeres

Centromere	Length of CENH3-Depleted Subdomains (kb)*^a^*	Length of H3k36me3 (kb)*^b^*	Length of H3k4me3 (kb)*^b^*	Length of H3k9ac (kb)*^b^*	Length of H3K27me3 (kb)*^b^*
*Cen2*	110 (10.4%)	4.6 (100%)	12.0 (90%)	13.8 (85%)	—
*Cen3*	128 (26.2%)	29.2 (100%)	1.6 (100%)	2.0 (77%)	—
*Cen4*	79 (15.6%)	0.8 (100%)	1.6 (62%)	1.6 (42%)	—
*Cen5*	125 (9.9%)	—	3.4 (100%)	1.8 (100%)	3.8 (100%)
*Cen8*	184 (23.4%)	—	—	—	—
*Cen9*	69 (17.9%)	—	—	—	—
*Cen10*	151 (13.3%)	21.8 (69%)	11.2 (100%)	4.6 (58%)	—

a Lengths of CENH3-depleted subdomains. Numbers in brackets are percentages of the CENH3-depleted subdomains in the centromeres.

b Lengths of histone modification peaks in CENH3-depleted subdomains. Numbers in brackets are the percentage of the modifications located at CENH3-depleted subdomains. “—” indicates no peaks identified in CENH3-depleted subdomains.

### Nucleosome positioning in maize centromeres

**Figure 3:**
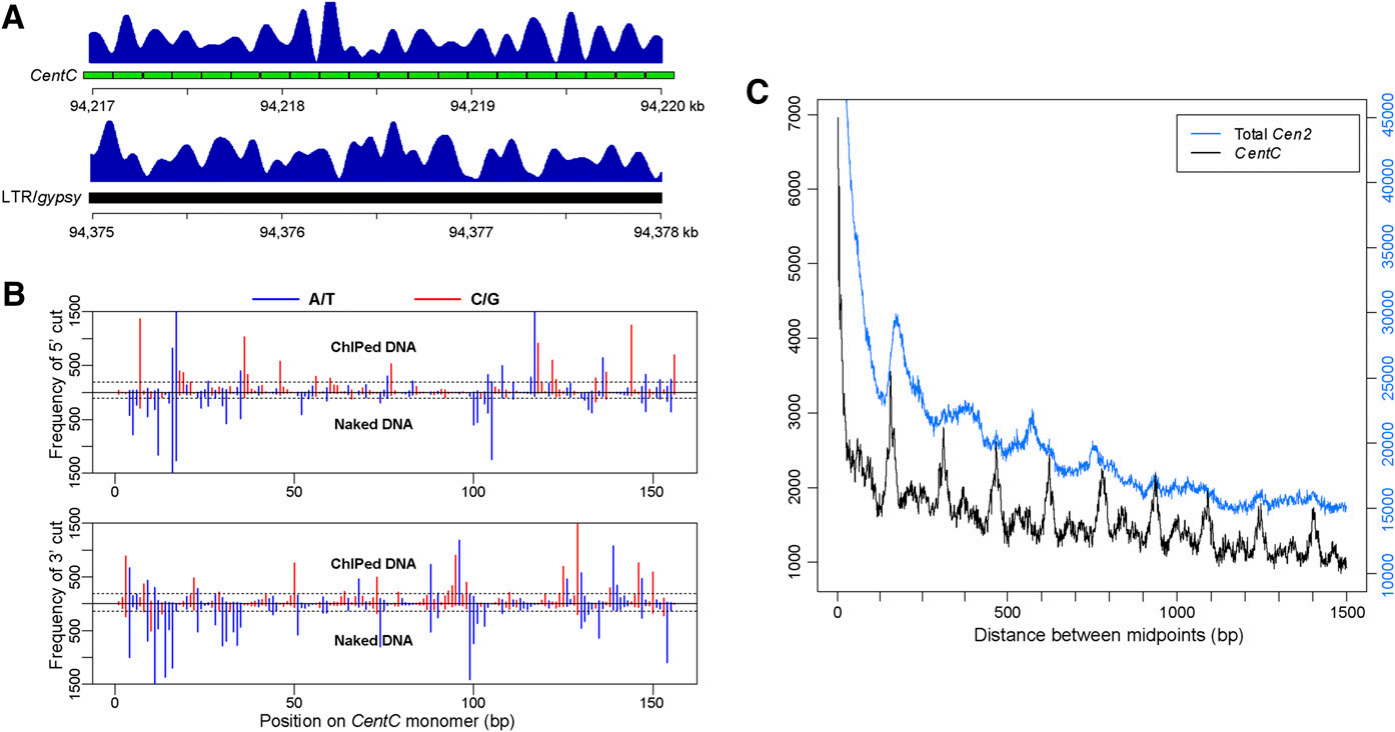
Positioning of CENH3 nucleosomes and MNase cutting patterns associated with *CentC* repeat. (A) Well-positioned CENH3 nucleosomes in *Cen2*, as indicated by CENH3 ChIP-seq reads. Top panel, a region containing an array of 20 *CentC* monomers marked by green bars. Most peaks were phased with individual *CentC* monomers. Bottom panel: a region containing *Ty3*/*gypsy* LTR retrotransposon sequences. Most CENH3 nucleosomes were well positioned but were not phased. (B) MNase cutting pattern on a consensus *CentC* repeat monomer. Vertical bars represent the frequency of cutting at each position on the *CentC* monomer. The *y*-axis shows the number of cuts in ChIPed DNA and naked DNA datasets. The *x*-axis shows each site of the consensus *CentC* monomer (156 bp). Black dashed lines mark the top 20% cutting frequency. (C) Phasogram of CENH3 nucleosomes in *Cen2*. Total *Cen2*: all fragments mapped to *Cen2* were used for the phasogram; *CentC*: only fragments mapped to *CentC* arrays were calculated for the phasogram. The *y*-axes is the frequency of distance between the midpoints of two fragments.

Although CENH3 ChIP-seq datasets were developed previously (Wolfgruber *et al.* 2009; Wang *et al.* 2014; Gent *et al.* 2015), we produced the highest volume of ChIP-seq reads from a specific maize genotype. This high volume of paired-end reads provided an opportunity to investigate the positioning of individual maize CENH3 nucleosomes. Each pair of sequence reads was first merged into a single DNA fragment, which provided information on the size and boundaries of individual DNA fragment that wrap single CENH3 nucleosomes. Approximately 93–95% of the paired reads were merged to a single fragment from the two libraries. Mapping merged fragments to the B73 genome revealed many sharp peaks in centromeres, which represent well-positioned CENH3 nucleosomes (Figure 3).

We observed sharp peaks associated with individual *CentC* monomers in *Cen2* (Figure 3A), indicating that CENH3 nucleosomes preferentially bind to *CentC* repeats such that the same histone-DNA contacts are formed; this phenomenon is termed nucleosome phasing and is related to innate sequence preferences of histones. Phasing of CENH3 on *CentC* arrays was further evidenced by phasograms of *CentC* arrays with a periodicity of approximately the same length as the *CentC* monomer ∼156 bp (Figure 3C**)**. In contrast, we did not detect a similar nucleosome phasing associated with non-*CentC* sequences in *Cen2*. For example, although positioned CENH3 nucleosomes were associated with retrotransposon-related sequences (Figure 3A), these nucleosomes did not show any specific periodicity as *CentC*-associated nucleosomes.

We next examined if the cutting site pattern associated with the *CentC* repeats was caused by sequence preference of MNase using a large sequence dataset derived from MNase-digested B73 genomic DNA. The distribution patterns of the cutting sites on the *CentC* repeat were clearly different between naked DNA and ChIPed DNA (Figure 3B). The cutting sites on naked *CentC* biased to A/T sites, consistent with the sequence preference of MNase (Horz and Altenburger 1981; Drew 1984; Telford and Stewart 1989). By contrast, the cutting sites of ChIPed DNA did not show A/T preferences (Figure 3B). Among the top 20% most frequently cut sites on the *CentC* repeat, 92% were A or T on naked DNA, but only 45% were A or T on ChIPed DNA.

### Transcription of genes in maize centromeres

CENH3-depleted subdomains in rice centromeres are occupied by canonical nucleosomes and contain active genes (Wu *et al.* 2011). We next investigated the transcriptional activity associated with maize centromeres. A total of 34 protein-coding genes were annotated in seven maize centromeres, of which 21 were located in CENH3-depleted subdomains, and 13 located in CENH3-enriched regions (Table S4). Eleven of the 21 genes (52%) in CENH3-depleted subdomains were expressed in shoot and/or root tissues based on RNA-seq datasets (Wang *et al.* 2009). In contrast, transcription was not detected for any of the 13 genes located in CENH3-enriched regions (Table S4). Six of 13 inactive genes located in the CENH3-enriched regions were shorter than 100 amino acids, and lack homologs (≥70% identity) in other plant species, suggesting that these are likely nonfunctional genes.

Maize centromeres also included a total of nine genes annotated as “low confidence genes” or “novel transcript” that lack homologs in other plant species, or lack evidence of transcription and/or intact coding capacity (Table S4). Analysis of RNA-seq datasets revealed potential transcription associated with eight of the low confidence genes as well as one “novel transcript” in *Cen2* (Table S4). Only two of these genes, which showed low levels of transcription (FPKM of 1.17 and 1.23, respectively) in roots (but not in leaf), were located in CENH3-enriched regions.

**Figure 4:**
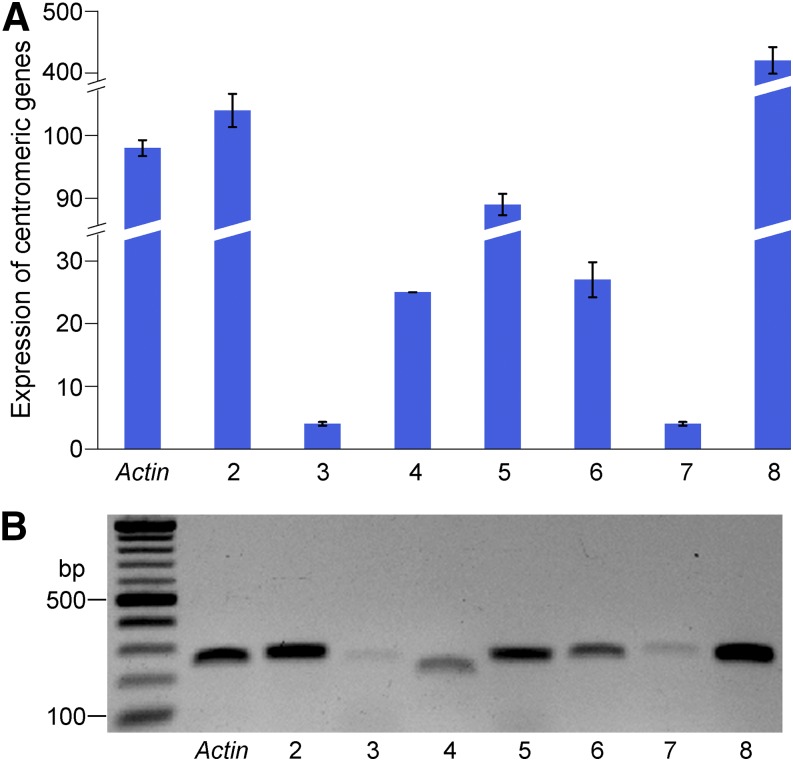
Validation of expression of centromeric genes in maize. (A) qRT-PCR validation of a set of seven centromeric genes. RNAs were isolated from 10-day-old seedlings of B73. The *y*-axis represents the relative expression level normalized by setting the expression of the *Actin* gene to 100. (B) SemiqRT-PCR analysis of the same set of centromeric genes. Validated genes include: Lane 2: *GRMZM2G040843* (FPKM 22.8); Lane 3: *GRMZM2G175425* (FPKM 1.1); Lane 4: *GRMZM2G137715* (FPKM 2.3); Lane 5: *GRMZM5G820434* (FPKM 5.6); Lane 6 *GRMZM2G071042* (FPKM 13.7); Lane 7: *GRMZM2G409893* (FPKM 5.7); and Lane 8: *GRMZM2G083935* (FPKM 3.0). Note: the levels of expression of each gene correlated in qRT-PCR and semiqRT-PCR experiments.

We selected seven centromeric genes to validate their transcription. These genes have different expression levels based on RNA-seq data. Transcription of all seven genes in seedlings was detected by both qRT-PCR and semiqRT-PCR, including genes *GRMZM2G175425* and *GRMZM2G137715* with FPKM 1.1 and 2.3, respectively (Figure 4). The expression of a low confidence gene *GRMZM5G820434* (FPKM 5.6) was also confirmed.

### Histone modifications associated with CENH3-depleted subdomains

We next investigated the histone modification patterns associated with maize centromeres. Genome-wide datasets of active histone marks H3K4me3, H3K36me3, and H3K9ac, and repressive histone mark H3K27me3 were developed using maize shoot tissue (Wang *et al.* 2009). We identified 64 peaks in seven centromeres (see *Materials and Methods*), which were associated with one to four of these histone marks (Table S5). Most of these peaks (62 of 64) were associated with the three active marks, and only two peaks associated with the repressive mark H3K27me3. In addition, most peaks (54 of 64) were found in CENH3-depleted subdomains. Interestingly, 47 of the 54 peaks located in CENH3-depleted subdomains were associated with annotated genes (Table S5). H3K36me3 was the most prevalent modification found in the CENH3-depleted subdomains (Table 2). Of the 20 transcribed centromeric genes, 17 (85%) were associated with active histone modifications (Figure 2 and Table S5). H3K4me3 and H3K9ac are generally enriched around transcription start sites of most maize genes. By contrast, H3K36me3 is more broadly associated with gene bodies (Figure S2). Thus, the centromeric genes showed similar histone modification patterns as most maize genes (Figure S2). These results indicate that CENH3-depleted subdomains in maize centromeres are occupied by canonical nucleosomes and share similar chromatin properties with euchromatic regions in the maize genome.

**Figure 5:**
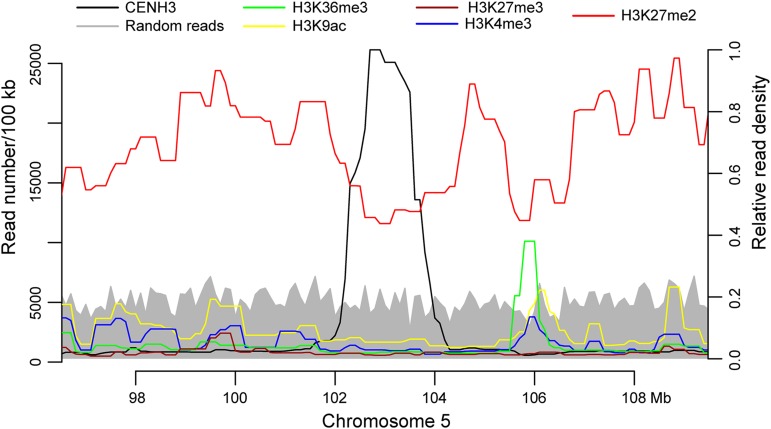
Distribution of sequencing reads associated with histone modifications and CENH3 in the centromeric and pericentromeric regions of maize chromosome 5. Chromosome 5 was divided into 100-kb windows, and read numbers of CENH3, H3K4me3, H3K27me2, H3K9ac, H3K27me3, H3K36me3 and randomly generated 150-bp reads were calculated in each window. Note the depletion of H3K27me2 within *Cen5* as compared to the pericentromeric region. The left *y*-axis represents the numbers of CENH3 ChIP-seq and random reads in each 100-kb window. The right *y*-axis represents the relative read density associated with different histone modifications.

H3K27me2 is a heterochromatic histone mark and is enriched with repetitive elements in the maize genome (Shi and Dawe 2006; Gent *et al.* 2014). Interestingly, H3K27me2 was generally depleted in centromeres compared to the flanking pericentromeric regions (Figure 5 and Figure S3). The reduced H3K27me2 in centromeres relative to pericentromeres was not due to mappability of the sequences in centromeric regions because there was no mapping bias of random reads generated from the B73 genome (see *Materials and Methods*). These results were also in agreement with previous cytological studies, in which H3K27me2 signals were detectable in centromeres, but were relatively weaker than in pericentromeric regions (Shi and Dawe 2006; Jin *et al.* 2008). In addition, H3K27me2 was also significantly reduced in the CENH3-enriched subdomains relative to the CENH3-depleted subdomains (Kolmogorov-Smirnov test, one-tailed) (Figure S4).

### Chromatin features associated with expanded maize centromeres in the genetic background of oat

The CENH3-binding domains of maize centromeres expand from ∼1.8 Mb to 3.6 Mb in the genetic background of oat to adapt to a similar size as oat centromeres (Wang *et al.* 2014). Interestingly, several centromeres, including *Cen2*, *Cen5*, *Cen9*, and *Cen10*, expand exclusively toward a single direction, either in the short or in the long arm, suggesting that the expansion direction is not random. The expanded regions appear to be relatively deficient for active genes (Wang *et al.* 2014).

**Figure 6:**
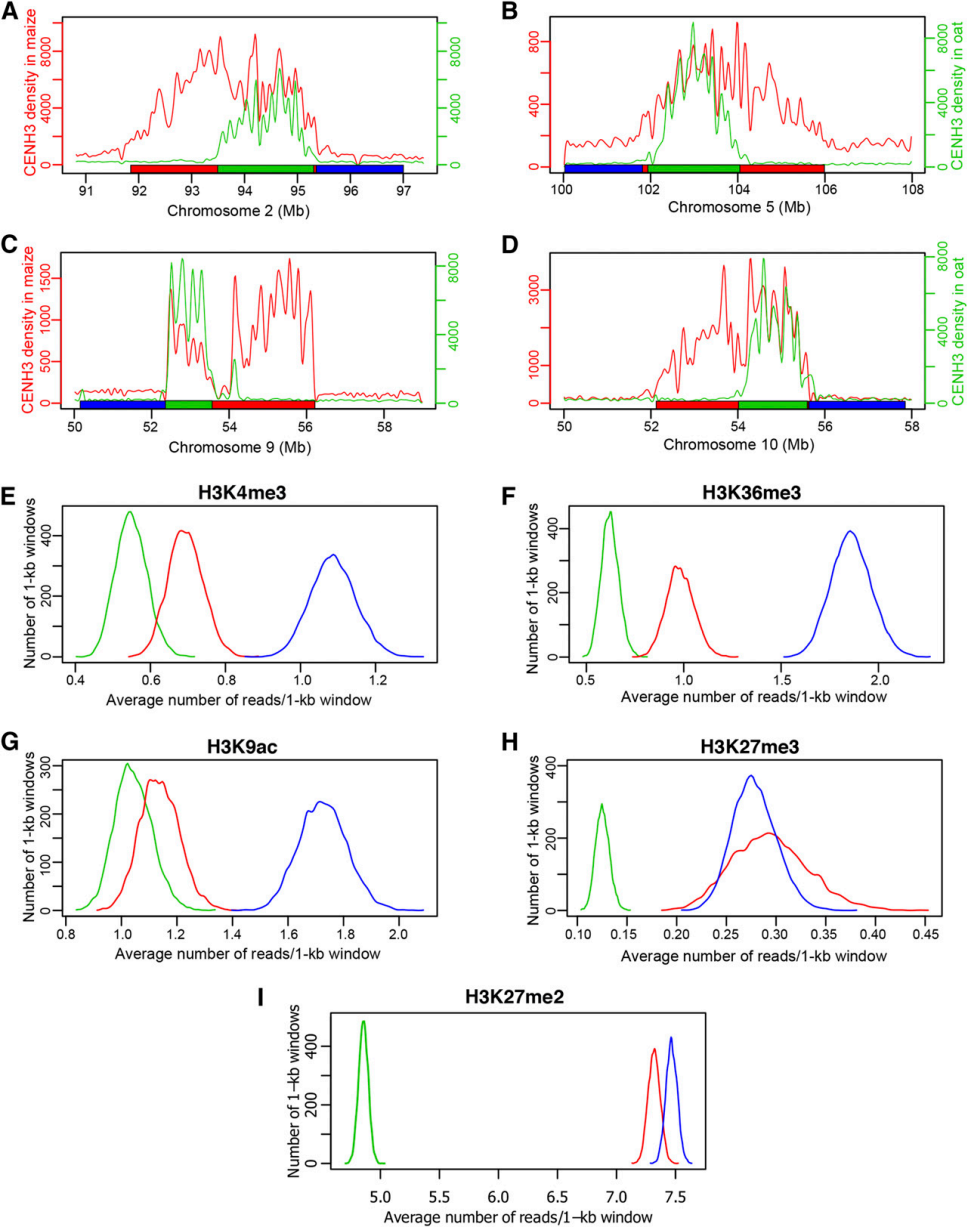
Centromere expansion and histone modifications. (A–D) Diagrammatic illustrations of expansion of maize *Cen2*, *Cen5*, *Cen9*, and *Cen10*, respectively, in the genetic background of oat (Wang *et al.* 2014). Green lines represent CENH3 ChIP-seq read density in maize, red lines represent CENH3 ChIP-seq read density in oat. Green horizontal bars show the locations of the four centromeres in maize. Red horizontal bars show the locations of expanded regions in oat. Each blue horizontal bar represents a nonexpanded region of the same size as the red bar of the same centromere. (E–I) Quantification of H3K4me3, H3K36me3, H3K9ac, H3K27me3, and H3K27me2, respectively, in original maize centromeres (green lines), expanded centromeric regions in oat (red lines) and nonexpanded regions (blue lines). The histone modification data are presented as the average of four centromeres (*Cen2*, *Cen5*, *Cen9*, and *Cen10*). The *y*-axis represents the number of 1-kb windows. The *x*-axis represents the average number of reads in each 1-kb window.

To further explore the potential barriers that prohibit maize centromere expansion in oat background, we compared the histone modification patterns associated with the expanded region flanking one side of the original centromere (Figure 6, A–D, regions marked by red bars) with the patterns associated with nonexpanded region flanking the other side of the centromere (Figure 6, A–D, region marked by blue bars). We analyzed the average numbers of ChIP-seq reads, using in 1-kb windows, of H3K4me3, H3K36me3, and H3K9ac and H3K27me3 associated with expanded and nonexpanded regions in four centromeres (*Cen2 Cen5*, *Cen9*, and *Cen10*). The expanded regions contained significantly lower numbers of reads (*p*-value < 1 × 10^–5^, two-tailed) of H3K4me3, H3K36me3, and H3K9ac than nonexpanded regions (Figure 6, E–G). Thus, the expanded regions were less euchromatic than the nonexpanded region. A similar read density of H3K27me3 was observed in expanded and nonexpanded region (*p*-value = 0.69, two-tailed), probably due to the lack of H3K27me3 in the pericentromeric regions (Figure 6H). We also found similar read density of a heterochromatin mark H3K27me2 in expanded and nonexpanded regions (*p*-value = 0.50, two-tailed) (Figure 6I), suggesting that the heterochromatin marked by H3K27me2 is not likely to play a role in the one-directional expansion of the centromeres.

## Discussion

We determined patterns of maize CENH3 nucleosome occupancy across centromeres by combining CENH3 ChIP and deep sequencing. Consistent with previous studies in rice (Yan *et al.* 2008; Zhang *et al.* 2013), we found that regions with high CENH3 enrichment were interspersed with regions of low CENH3 enrichment, at least some of which contain expressed genes. Although the chromatin environment of centromeres differs from the rest of the genome (Verdaasdonk and Bloom 2011), we found that expressed genes located in centromeres exhibit histone modification associated with typical active genes, including H3K36me3, H3K4me2, and H3K9ac (Figure S2). The fact that expressed genes in centromeres have lower CENH3 enrichment than other parts of centromeres suggests that transcription may inhibit CENH3 accumulation. We also speculate that very high levels of CENH3 within genes would be incompatible with transcription because of a different N-terminal tail structure that cannot be modified with the H3 modifications associated with transcription regulation (Gassmann *et al.* 2012; Wang *et al.* 2014). Regardless of how CENH3 affects or is affected by transcription, it is clear that the expressed genes can exist inside of functional centromeres in maize.

Comparison of CENH3 nucleosome distribution in maize and rice reveals several interesting differences. First, maize centromeres are much larger than rice centromeres (∼2 Mb *vs.* ∼750 kb). Second, maize centromeres appear to have greater CENH3 nucleosome occupancy across their centromeres: 87% of maize centromeres were made of domains of high CENH3 enrichment, compared with 38–49% of rice centromeres (Yan *et al.* 2008). These numbers should be interpreted with caution, however, as the rice study measured enrichment using a normalization-free method that does not allow precise measurements of CENH3 enrichment. Further supporting evidence for greater CENH3 nucleosome occupancy in maize centromeres than in rice comes from the different numbers of genes in centromeres in each species: while maize centromeres contain from one to eight genes in each centromere, rice centromeres contain from 17 to 21 genes (Yan *et al.* 2008).

High occupancy of CENH3 nucleosomes in maize is not limited to regions containing the *CentC* repeats, nor is it an artifact from mapping reads to areas of low sequence complexity. First, *Cen2* and *Cen5* contain the lowest amounts of *CentC* repeats among B73 centromeres (Albert *et al.* 2010), but regions of high CENH3 enrichment make up nearly 90% of these two centromeres. Second, *CentC* and the two dominant centromeric retrotransposons *CRM1* and *CRM2* of maize are highly enriched for CENH3 nucleosomes (Zhong *et al.* 2002; Jin *et al.* 2004; Wolfgruber *et al.* 2009; Gent *et al.* 2011). Differences in centromere size and CENH3 abundance between maize and rice may be a consequence of maize having much a larger genome and chromosome size. An elegant immunofluorescence-based comparison of centromere and genome size revealed that centromere size correlates with genome size among the genetically related grass species, suggesting large amounts of cenH3 benefit the stabilization of the spindle and separation of large chromosomes (Zhang and Dawe 2012), Based on these data, we propose that abundant CENH3 nucleosomes are important for centromere function in species with large chromosomes, and we predict that characterization of additional large-chromosome species will reveal maize-like centromeres that are either large or have a high percentage of CENH3-enriched subdomain within the centromeres.

Consistent with prior studies of nucleosome organization for the maize centromeric repetitive elements *CentC*, *CRM1*, and *CRM2* (Gent *et al.* 2011), we found that the dominant pattern of nucleosome spacing across centromeres revealed by micrococcal nuclease digestion was approximately 190 bp between nucleosome midpoints (Figure 3C). However, we also found evidence for reproducible positioning, or phasing, of nucleosomes relative to the *CentC* repeat (Figure 3A). Phasing of CENH3 nucleosomes on the 156-bp *CentC* repeats mirrors the recently reported phasing of cenH3 nucleosomes along the 155-bp *CentO* repeats in rice (Zhang *et al.* 2013). Thus, nucleosome phasing mediated by centromeric tandem repeats may be a common mechanism that contributes toward centromere/kinetochore function. The existence of distinct fragment sizes following MNase digestion in both input and ChIP (Figure 1) could reflect flexibility of DNA wrapping around cenH3 nucleosomes (Hasson *et al.* 2013). Another potential explanation for smaller DNA fragments is that nucleosomes are not limited to the classical eight-histone and 147-bp wrapping DNA structure, and such alternative histone complexes are particularly abundant among cenH3 containing nucleosomes. Indeed, multiple experimental approaches in diverse species have indicated that cenH3 nucleosomes can exist in a complex of histone tetramers rather than the canonical histone octamer (Dalal *et al.* 2007; Dimitriadis *et al.* 2010; Shivaraju *et al.* 2012).

Centromeric chromatin is associated with distinct histone modification patterns as compared to classical euchromatin and heterochromatin in several model eukaryotes. In humans and *Drosophila melanogaster*, CENP-A nucleosomes are interspersed with H3 nucleosomes marked by H3K4me2. These H3 nucleosomes are associated neither with the euchromatic mark H3K4me3 nor with the heterochromatic marks H3K9me2 and H3K9me3 (Sullivan and Karpen 2004). H3 nucleosomes marked with H3K4me were also detected in *S. pombe* centromeres (Cam *et al.* 2005). However, this distinct association of H3K4me2 with centromeres was not found in rice centromeres. Transcribed genes in rice centromeres, however, were associated with classical euchromatic histone modifications marks, including H3K4me2, H3K4me3, H3K36me3, and H3K4ac (Wu *et al.* 2011). We detected a similar association of euchromatic histone modifications with active genes in maize centromeres (Figure 2). Maize centromeres had reduced levels of the heterochromatic mark H3K27me2 relative to pericentromeric regions (Figure 5 and Figure S3), which agrees with cytological observations that maize centromeres were weakly stained with immunofluorescence from heterochromatic histone modification marks (Shi and Dawe 2006; Jin *et al.* 2008). Thus, maize centromeres appear to be dominated by subdomains enriched with CENH3 and have a relative depletion of histone modifications associated with H3 histone. We find no evidence that histone H3 modifications studied in this work serve as a major epigenetic mark for centromere identity.
